# *In vivo* insights into irregular voice production as a complex nonlinear system—a case study

**DOI:** 10.1098/rsif.2025.0086

**Published:** 2025-08-13

**Authors:** Louisa Traser, Mario Fleischer, Daniel Priegnitz, Fiona Stritt, Marie Köberlein, Jonas Kirsch, Johannes Fischer, Hanspeter Herzel, Dirk Mürbe, Bernhard Richter, Matthias Echternach

**Affiliations:** ^1^ Freiburg Institute for Musicians‘ Medicine, University of Music Freiburg, Medical Center – University of Freiburg, Freiburg Centre for Music Research and Teaching, Freiburg, Germany; ^2^ Faculty of Medicine, University of Freiburg, Freiburg, Germany; ^3^ Department of Audiology and Phoniatrics, Charité-Universitätsmedizin Berlin, Corporate Member of Freie Universität Berlin and Humboldt-Universität zu Berlin, Berlin, Germany; ^4^ https://www.vocalsontherocks.de/, Freiburg, Germany; ^5^ Department of Otorhinolaryngology, Division of Phoniatrics and Pediatric Audiology, Ludwig-Maximilians-Universität München Medizinische Fakultät, München, Bayern, Germany; ^6^ Department of Medical Physics, Medical Center—University of Freiburg Department of Radiology, Freiburg, Baden-Württemberg, Germany; ^7^ Institute for Theoretical Biology, Charité and Humboldt-University, Humboldt University of Berlin Institute for Theoretical Biology, Berlin, Germany

**Keywords:** irregular singing, nonlinear dynamics, period-doubling oscillation, vocal ventricular phonation

## Abstract

The human voice exhibits diverse expressions, primarily generated by the regular oscillatory movement of both vocal folds. However, further vocal tract structures can also oscillate, as observed in different singing styles, supraglottic voice substitutes and functional voice disorders. To describe the spectrum of irregular phonation types from a nonlinear systems perspective, we conducted *in vivo* investigations with a professional metal singer using transnasal high-speed digital imaging combined with electroglottography and acoustic data. Here, we analysed 10 distinct voice qualities. Some of these show oscillatory mechanisms characterized by nearly periodic patterns that differ in three key aspects: the ratio of glottal to supraglottic oscillations, their behaviour during the glissando and the oscillating structures primarily involved. Both reduced glottal adduction and increased glottal and supraglottic compression contribute to a greater degree of irregularity. The supraglottic oscillations can coexist with various glottal oscillation mechanisms. Based on the *in vivo* data, we identified various nonlinear phenomena of different categories that include synchronized oscillations with defined frequency ratios, bifurcations, biphonations and chaotic oscillations.

## 1. Introduction

The human voice is essential for social and cultural interactions. It offers a broad portfolio of expressions, ranging from whispering to screaming and various singing styles.

Normal voice production is characterized by a regular, oscillatory movement of both vocal folds (VF), driven by the subglottic pressure. In the classic linear source-filter theory, the VF oscillation modulates the glottal airflow into a pulsating flow. This flow is the basis of the sound formation, which is then ‘filtered’ by resonances of the air-filled space between VFs and mouth opening, the vocal tract [1].

Supraglottic structures, such as the ventricular folds (VenF), the aryepiglottic folds, the arytenoids or the epiglottis, influence the voice signal during usual voice production in different ways: (i) modification of vocal tract configuration changes the transfer function and thus the ‘filter’ of the primary voice signal; and (ii) by so-called source–filter interactions, in which the configuration of the vocal tract influences VF vibration by reflecting sound. It was shown that a specific geometrical positioning of the VenF can either facilitate or impede the phonation: a slight VenF adduction amplifies the magnitude of glottal oscillations. When VenF adduction increases, beyond a certain point, the relationship reverses [2,3].

Other vocal tract structures can oscillate in analogy to the VFs. The VenF are the most frequently described structure that can be involved as an oscillator in the generation of a sound source. However, these folds, primarily composed of glands and fat and located cranially to the VFs, are less structurally complex, missing a layered composition [4]. VF and VenF can oscillate simultaneously, denoted as vocal–ventricular fold co-oscillations as first described by Fuks *et al.* [5]. Such mechanisms have been documented in Asian and Sardinian throat singing [6] as well as in rock/metal singing [7]. In terms of nonlinear dynamics, the vocal–ventricular fold co-oscillations can be regarded as coupled oscillator dynamics, since the VF and VenF can alone induce self-sustained oscillations, and they interact with each other aerodynamically [8].

While the VF–VenF co-oscillation mechanisms during throat singing are characterized by some degree of periodicity [6,7], there are also unintended VenF oscillations that result in an irregular voice production and are therefore described within the context of ventricular dysphonia [9,10]. However, there are conditions where supraglottic oscillation, even if irregular, is desirable. As it can be a more relevant sound source than the VFs, that is particularly relevant when VF oscillation is insufficient for voice production. In such cases, the VFs are often structurally compromised, resulting in a partial or complete loss of their vibratory function.

Reasons for the damage of the VF could be, among others, scarring and lesions or invasive growth, as well as a result of surgical removal of parts of the whole VFs or partial laryngectomies for malignant diseases. After treatment of the primary disease, the focus is often on restoring an efficient voice. This is particularly important for long-term quality of life, as voice disorders are associated with comorbidities in the area of affective disorders such as depression [11]. In addition to surgical methods for improving vocal function, learning supraglottic replacement mechanisms is a potential avenue for voice rehabilitation.

For this reason, it may be worthwhile to gain a deeper understanding of supraglottic voice production mechanisms in order to more effectively transfer these principles to functional voice rehabilitation. In the past, studies employed the framework of nonlinear dynamic systems to characterize a vocal–ventricular fold system through both physical experimentation and computational modelling [8,12]. Their findings elucidate that a VF–VenF co-oscillation system can be conceptualized as two interlinked oscillators, exhibiting diverse cooperative behaviours. These behaviours encompass synchronized oscillations with defined frequency ratios, as well as desynchronized oscillations leading to chaotic dynamics.

While these experimental models have traditionally focused solely on the VF and VenF, *in vivo* observations highlight the involvement of additional oscillators such as the epiglottis or arytenoid cartilages in human communication and artistic expression.

To comprehensively describe various irregular voice production mechanisms, including the diversity of different supraglottic oscillators from a nonlinear systems’ perspective, we conducted *in vivo* investigations with a professional singer and vocal coach. We aimed to capture a wide spectrum of vocal behaviours, which include supraglottic oscillation, using transnasal high-speed digital imaging in combination with electroglottography and acoustic data.

Enhancing our comprehension of these intricate systems holds promise not only for refining voice pedagogic practices in artistic domains but also for illuminating pathophysiological links.

## 2. Methods

### 2.1. Participant and tasks

According to the declaration of Helsinki and with the local ethic committee (Medical Ethics Committee of the University of Munich no. 18/769), we analysed the phonatory outcome of one male participant, professional singer and singing teacher (co-author D.P.). He vocalized the vowel /i:/ on 10 different irregular voice qualities. If possible the singer was asked to crossfade from his subjectively normal (denoted as ‘clear’) phonation to the respective irregular voice quality. Then, he was asked to perform a glissando over the whole comfortable range of the respective quality if possible. He was asked to sustain both the lowest and the highest pitch for at least 1 s (later referred to as low and high stable phonation, respectively). For the qualities that did not allow for pitch control or where the singer did not consciously perceive any pitch, the singer was asked to phonate the quality in a way that was typical for him in an artistic sense.

All measurements were taken in a seated, upright position. The vowel /i:/ was chosen for all tasks to ensure the best possible view of the larynx, even if this is not favoured for certain qualities from a voice pedagogic perspective.

It should be noted that the nomenclature of the qualities we used throughout the manuscript followed the nomenclature of the singer, as no generally accepted nomenclature exists. The pitch was determined by the singer from the audio for the different qualities, which can be taken from table 1.

**Table 1.** Summarized results of perceived note/pitch (corresponding to Hz) and $f_{\text{o}}$ (including standard deviation) in iEGG, audio, SAW and GAW signals for all voice qualities.

group	quality	note/pitch in Hz	$f_{\text{o}}$ in Hz (iEGG)	$f_{\text{o}}$ in Hz (Audio)	$f_{\text{o}}$ in Hz (SAW)	$f_{\text{o}}$ in Hz (GAW)
1	Undertone	C3/131	68 ± 0	68 ± 0	68 ± 0	—
1	Undertone	F3/175	89 ± 0	89 ± 0	89 ± 1	—
1	Distortion	A3/220	216 ± 3	108 ± 1	108 ± 2	216 ± 3
1	Distortion	Db4/277	277 ± 2	277 ± 2	56 ± 0	277 ± 2
1	Growl	Eb3/156	77 ± 1	77 ± 1	77 ± 1	—
1	Growl	Bb3/233	77 ± 0	77 ± 0	77 ± 1	—
1	Rattle	Eb4/311	76 ± 1	76 ± 1	79 ± 3	—
1	Rattle	Bb4/466	67 ± 1	74 ± 6	67 ± 3	—
2a	VocalFry	−/−	45 ± 3	44 ± 2	—	44 ± 1
2a	Grunt	atonal/−	—	—	—	62 ± 8
2b	DeathGrowl	atonal/−	—	—	97 ± 5	—
2b	DeathShout	atonal/−	126 ± 5	—	127 ± 7	—
2b	FfullDistortion	atonal/−	72 ± 21	—	88 ± 29	—
2b	FryScream	atonal/−	—	—	—	—

### 2.2. Measurements

For all tasks, we acquired pictorial data during transnasal endoscopy of the larynx via high-speed videoendoscopy (HSV) (Fastcam SA-X2; Photron, Tokyo, Japan and ENF GP; Fa. Olympus, Hamburg, Germany; frame rate 20 000 fps, spatial resolution of 386 × 320 pixels, greyscale). Simultaneously, we recorded the acoustic pressure (audio signal) via microphone (DPA 4061, DPA microphones, Alleroed, Denmark) and the electroglottographic signal (EGG) (EG2-PCX2; Glottal Enterprises, Syracuse, NY, USA) as described before in [13] using an NI Interface (National Instruments, USB−6251, BNC, Austin, TX, USA). The audio signal was calibrated with regard to sound pressure level using a sound level meter (Tecpel, Sound Level Meter Datalogger dB measurement DSL331) and the Sopran software (Svante Granqvist, Karolinska, Stockholm, Sweden). The audio, EGG and HSV data were collected with a sampling frequency of *F*_s_ = 20 kHz. To achieve a consistent presentation of the open VF and the open VenF, we multiplied the EGG signal by ‘−1’. The orientation of the resulting sign-inverted EGG signal, i.e. iEGG = −1 ⋅ EGG is similar to the glottal and supraglottic area waveform (§2.3), i.e. a positive value of the glottal or supraglottic area waveform corresponds to a positive value of iEGG. Thus, it represents the inverted VF contact area [14] and [15, p. 419], peaking at the moment of minimal VF contact and reaching its minimum when the VF contact area is maximal.

### 2.3. Segmentation and post-processing of pictural data

In analogy to the established glottal area waveform (GAW) [16], denoting the time-dependent opening area at the level of the VFs, we segmented the supraglottic area waveform (SAW). For this purpose, the image material was adjusted in contrast and greyscale recognition threshold to minimize the visibility of VF oscillation as much as possible. As a result, the segmentation algorithm now detects primarily the supraglottic area instead of the glottal area. It is therefore important to mention that SAW encompasses various vibratory areas without distinguishing between them. Since we derived the SAW signal from two-dimensional imaging data, it cannot, unlike the iEGG signal, provide information about relative contact areas. Therefore, the minimum of the SAW signal does not necessarily correspond to the point of maximal supraglottic contact. The HSV videos were post-processed by means of rotation, fast-Fourier treatment and cropping as described in [17]. For voice qualities that at least temporarily allowed an unrestricted view of the VF vibrations, the classical GAW was additionally segmented.

For the visual analysis of the HSV data, videos from representative segments of both the low and high sustained phonation were created, each covering 100 ms and played back at 30 fps. For those voice qualities that could not be assigned to a pitch in subjective evaluation, this was established from a representative section in the middle of phonation. Additionally, the iEGG, SAW and/or GAW data are attached to the respective videos. Considering qualities in our data, where GAW and iEGG signals were available, we found no special attention regarding shifting the iEGG signal was needed [14].

### 2.4. Signal processing

All three measured non-audio metrics (iEGG, GAW and SAW) were normalized by its maximum absolute value, to be optimally prepared for the usage of libraries restricted to audio(-like) signals. For the evaluation of the fundamental frequencies ($f_{\text{o}}$) and the short-term Fourier spectrograms (STFT), we used the probabilistic YIN algorithm [18], implemented in the library ‘librosa’ (v. 0.10.1 [19]). Plausibility check has been done by visual inspection of the $f_{\text{o}}$ traces overlaid on the STFT plots.

To detect frequency jumps from clear to irregular phonation, we carefully inspected the spectral properties of the Hann-windowed audio signals in Audacity (v. 2.4.2, https://www.audacityteam.org/) using a window length of 2048 samples.

### 2.5. Phase synchrony

Inspired by but not identical to Pedersen *et al.* [20], we computed the phase synchrony (PS) between iEGG and SAW to


(2.1) $$\begin{array}{rl} PS_{\text{iEGG/SAW}} & =\cos(|∠(\text{iEGG}+jH(\text{iEGG}))\\ & -∠(SAW+jH(SAW))|), \end{array}$$

where H denotes the Hilbert transform and ∠ is the angle of the analytic signal.

To detect time dependency of PS, we computed the band-pass filtered and segmented PS (we chose a variable window length $N_{\text{win}}=F_{\text{s}}/f_{\text{o}}^{\text{ min}}$, were $f_{\text{o}}^{\text{ min}}=0.2\cdot \text{min}\;[f_{\text{o}}^{\text{ iEGG}},f_{\text{o}}^{\text{ audio}}]$ for group 1 (see below) and $f_{\text{o}}^{\text{ min}}=20$ Hz for group 2). PS spans the range between ‘−1’ (phase shift of 180°) and ‘+1’ (phase shift of 0°, i.e. in-phase), whereas ‘0’ means that the two signals are either of identical frequency and phase shifted by 90° or the ratio of the (prominent) frequencies of the signals is an integer variable. We finally report medians, 25% and 75% quantiles (the difference of these quartiles is defined as the interquartile range (IQR)) of PS for each window). It should be noted that for a median of PS equal to zero and an IQR = 0, the two signals are of identical frequency and phase shifted by 90°.

All measured data and Python scripts used for analysis, computation and visualization are in the electronic supplementary material.

## 3. Results and discussion

Ten irregular voice qualities exhibited by a professional metal singer were analysed using HSV, iEGG and audio signals to delineate their underlying principles of voice production. We identified multiple oscillators associated with distinct laryngeal and pharyngeal sphincter mechanisms, oscillating either synchronized or independently, displaying quasi-periodic, periodic (also referred to as toroidal) or chaotic properties. These mechanisms can be considered complex systems within the framework of nonlinear physics due to bifurcations, jumps in pitch and subharmonics.

We found physiological reasons to categorize them into two groups with subgroups based on the categorization of VenF movements suggested by Bailly *et al.* [2].

In group 1, phonation types showed quasi-periodic oscillations of glottal and supraglottic structures with varying ratios of oscillation frequencies (rapid oscillatory motions with regular vibration according to [2]). The singer could control a subjective pitch with the techniques grouped here and actively alter it in a specific frequency range to phonate a glissando.

In group 2, phonation types showed irregular (from slight irregular to chaotic) glottal and supraglottic oscillations with various combinations (rapid oscillatory VenF motions with irregular vibration according to [2]). The singer could not actively control the pitch in the techniques in group 2. For these mechanisms, we further classified group 2 into:

— group 2a: the glottis was visible via HSV; and
— group 2b: the glottis was not visible because of supraglottic constriction.

In §3, the results are presented and evaluated in the context of the existing literature, considering each voice quality separately.

### 3.1. Group 1

All the mechanisms summarized here share the commonality of driving two oscillators that generate almost periodic vibrations. They differ in their ratio of glottal to supraglottic frequency evaluated in the electronic supplementary material, video Sl, the vibrating structures involved and the behaviour of the oscillators relative to each other during a conscious change in $f_{\text{o}}$. The oscillation ratio was constantly 2 : 1 or 1 : 1 during Undertone and Distortion. In contrast, during Growl and Rattle, the oscillation ratio changed during the glide (from 2 : 1 to 3 : 1 for Growl and from 4 : 1 to 7 : 1 for Rattle).

#### 3.1.1. Undertone

Undertone phonation involves oscillation of the VF and VenF in an oscillation ratio of 2 : 1 (electronic supplementary material, video SV01_Undertone_C3.mp4 and SV02_Undertone_F3.mp4). Supraglottic vibrations include the VenF, which exhibit the largest vibratory amplitudes and contact predominantly in their anterior sections for low stable phonation. In contrast, during high stable phonation, the whole VenF vibrated and were in complete contact. When visually accessible, VF vibration also exhibits complete closure.

A comparable mechanism has been described in the literature as vocal–ventricular–phonation, identified by Fuks *et al.* for Tibetan chant tradition [5]. Similar phonation types were also described for Mongolian Kargyraa [21,22] and Sardinian Bassu singing [23]. Notably, Bailly *et al.* [2] elucidate this mode within the context of ‘growly phonation’. It is important to note that the term ‘Growl’ is defined differently in our manuscript (§3.1.3), as is also done by, e.g. Sakakibara *et al.* [7].

**Figure 1.**
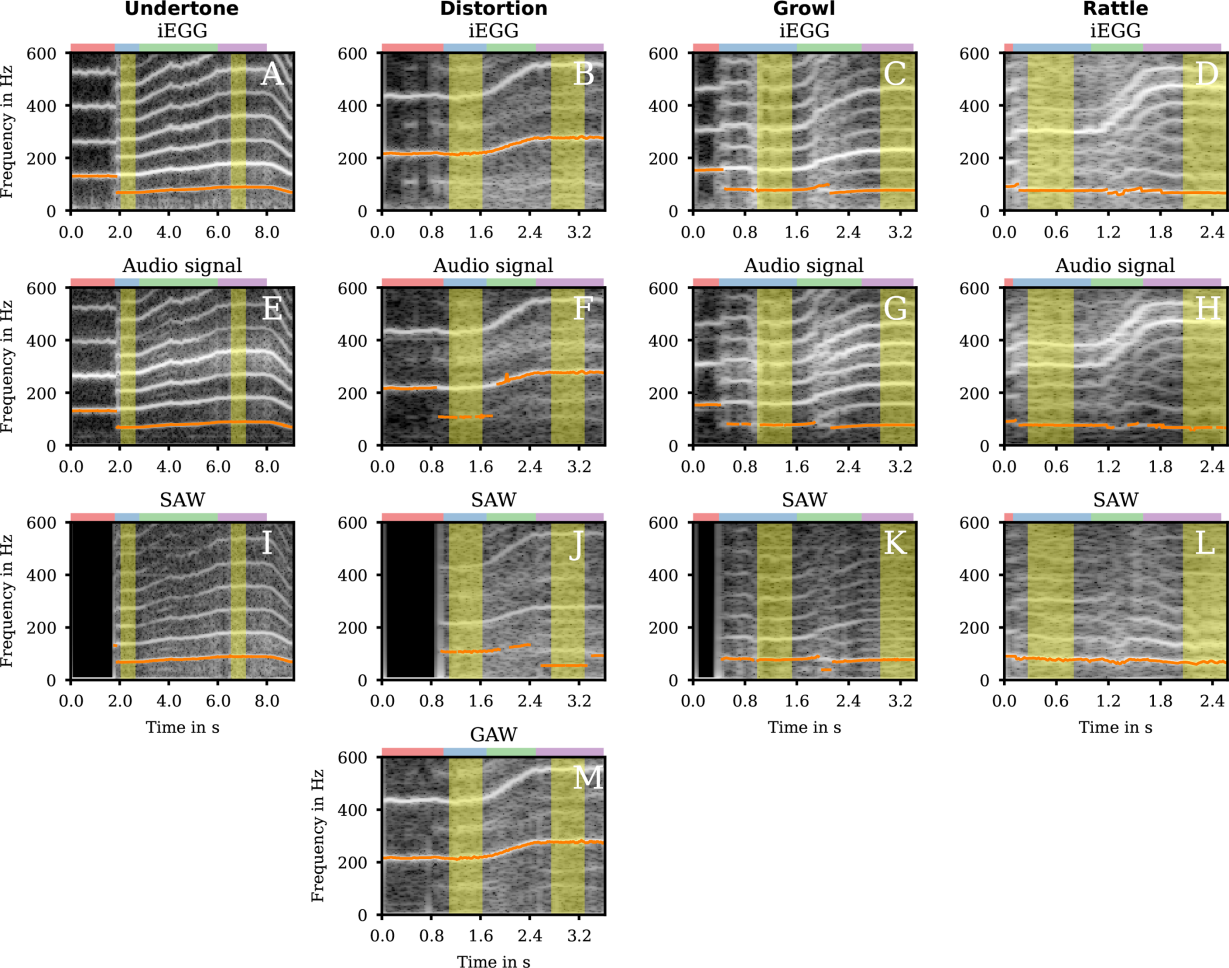
Spectrograms for group 1. The spectrograms of the iEGG signals (A–D), the audio signals (E–H), the SAWs (I–L) and GAW (M) for the qualities Undertone (1st column), Distortion (2nd column), Growl (3rd column) and Rattle (4th column) are shown in greyscale. The $f_{\text{o}}$ trajectories are symbolized in orange. At the top of each subfigure, the coloured rectangle denotes the region for clear phonation (red), low stable phonation (blue), glissando (green) and high stable phonation (purple). The areas highlighted in yellow correspond to the segments of sustained phonation depicted in the electronic supplementary material videos and analysed for their $f_{\text{o}}$ in table 1. Black regions in the SAW signal (I–K) indicate segments where the signal could not be obtained due to the absence of supraglottic oscillations.

During clear phonation, a $f_{\text{o}}$ of approximately 140 Hz is present in the iEGG and audio signal (figure 1A,E,I and electronic supplementary material, supplemental Undertone-hsdi_audio_glide.wav). At approximately 1.8 s, the singer switched to the Undertone quality, and a frequency jump from 128 to 137 Hz occurred. This indicates that the destabilization of the initial limit cycle by a subcritical bifurcation. Additional to this frequency jump, subharmonics, probably caused by period-doubling bifurcations, with $f_{\text{o}}$ of approximately 68 Hz (table 1), caused by supraglottic oscillations, occurred and remained during the glissando reaching about 89 Hz at high stable phonation (table 1). Additionally, during glissando between 3.2 and 4.2 s, further subharmonics, also probably caused by period-doubling bifurcations, are visible in the audio and SAW signal (figure 1E,I) and to a lesser extent also in the iEGG signal (figure 1A). This indicates that this nonlinear effect is mainly affected by supraglottic oscillations.

**Figure 2.**
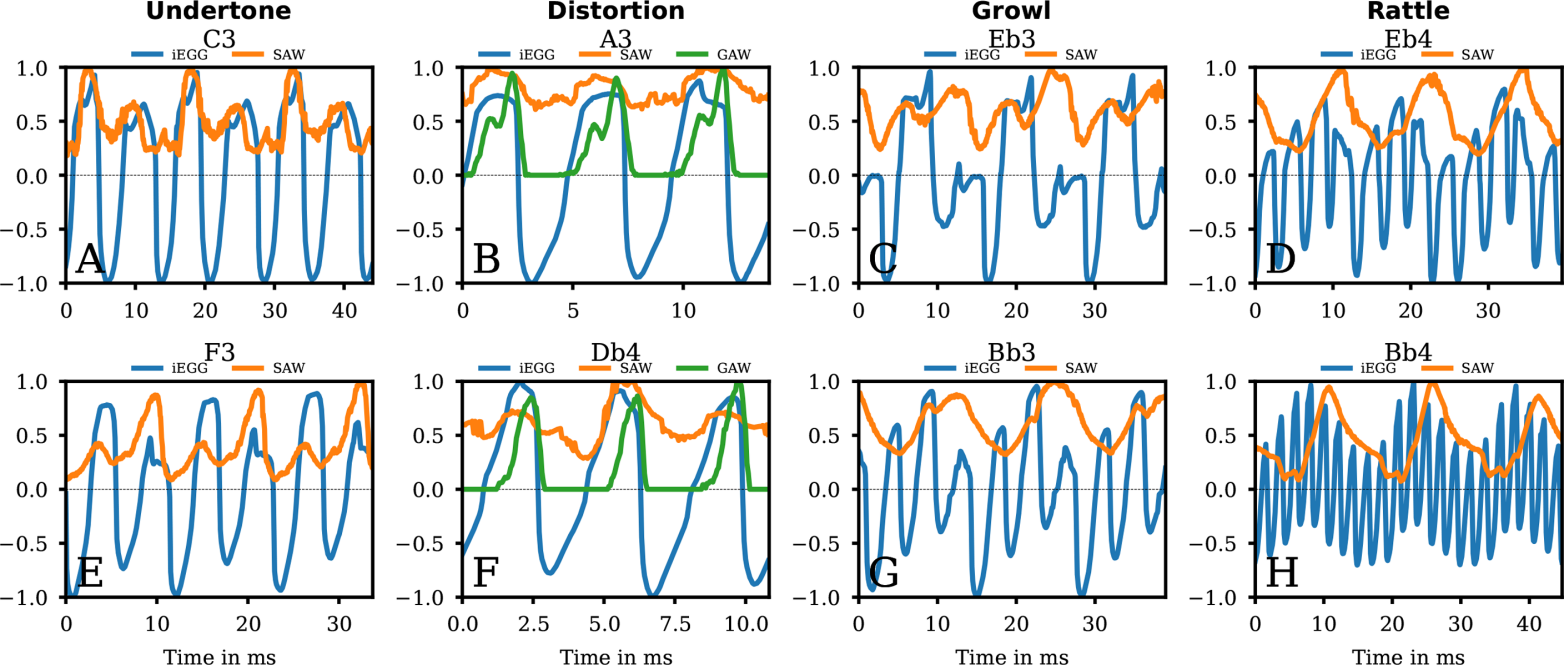
Detailed segments of signals of group 1. The iEGG, SAW and (if available) GAW signals as denoted in the legends for the qualities Undertone (A,E), Distortion (B,F), Growl (C,G) and Rattle (D,H). The first row corresponds to the low stable phonation whereas the second row corresponds to high stable phonation data (figure 1).

In detail, figure 2A,E displays three representative oscillation cycles for both the iEGG and SAW signals. During Undertone phonation, the SAW signal predominantly reflects the oscillatory movements of the VenF. SAW minima correspond to the maximum reduction of the supraglottic area due to the medial movement of the VenF. In contrast, SAW maxima correspond to the maximum expansion of the supraglottic area caused by their lateral movement. However, the oscillatory pattern of the VenF in the SAW signal does not exhibit a simple, consistent opening and closing motion over two VF vibration cycles (figure 2A,E). Instead, it displays a multi-peaked pattern, with additional minima simultaneously with the second VF closing phase (approximately at 8, 21 and 34 ms for C3 (figure 2A) and at 6, 17 and 28 ms for F3 (figure 2E)). Visually this relates to an additional oscillatory medial movement of VenF with a smaller amplitude and without VenF contact, which occurred between two main VenF closing movements (electronic supplementary material, video SV01_Undertone_C3.mp4 versus SV02_Undertone_F3.mp4). Our observations are in good agreement with [7,22] but, probably depending on the degree of VenF to VF adduction, slightly different from [5,6].

As a reminder, the iEGG signal represents the inverted vocal fold contact area, peaking at the moment of minimal VF contact and reaching its minimum when the VF contact area is maximal. However, previous studies on irregular phonation types have shown that the EGG (and consequently also the iEGG) signal can be further influenced by supraglottic vibrations [6,24]. These influences may occur either directly, through changes in VF oscillation, or indirectly, via alterations of the supraglottic impedance. Such factors could account for the varying amplitudes of the iEGG minima observed in our data (figure 2A,E), potentially due to an additional increase in conductivity during VenF contact.

In the presented data, $f_{\text{o}}$ of the iEGG, the audio and the SAW data continuously corresponded to approximately half of the perceived pitch of approximately 131 and 175 Hz, respectively (table 1). Within vocal–ventricular–phonation modalities, the supraglottic frequency often serves as a subjective correlate to perceived pitch. This observation aligns with the findings of Fuks *et al.* [5] and Sakakibara *et al.* [7], who documented suppression of every second VF pulse by the closure of the ventricular folds, thus influencing psychoacoustic perception towards a lower pitch.

**Figure 3.**
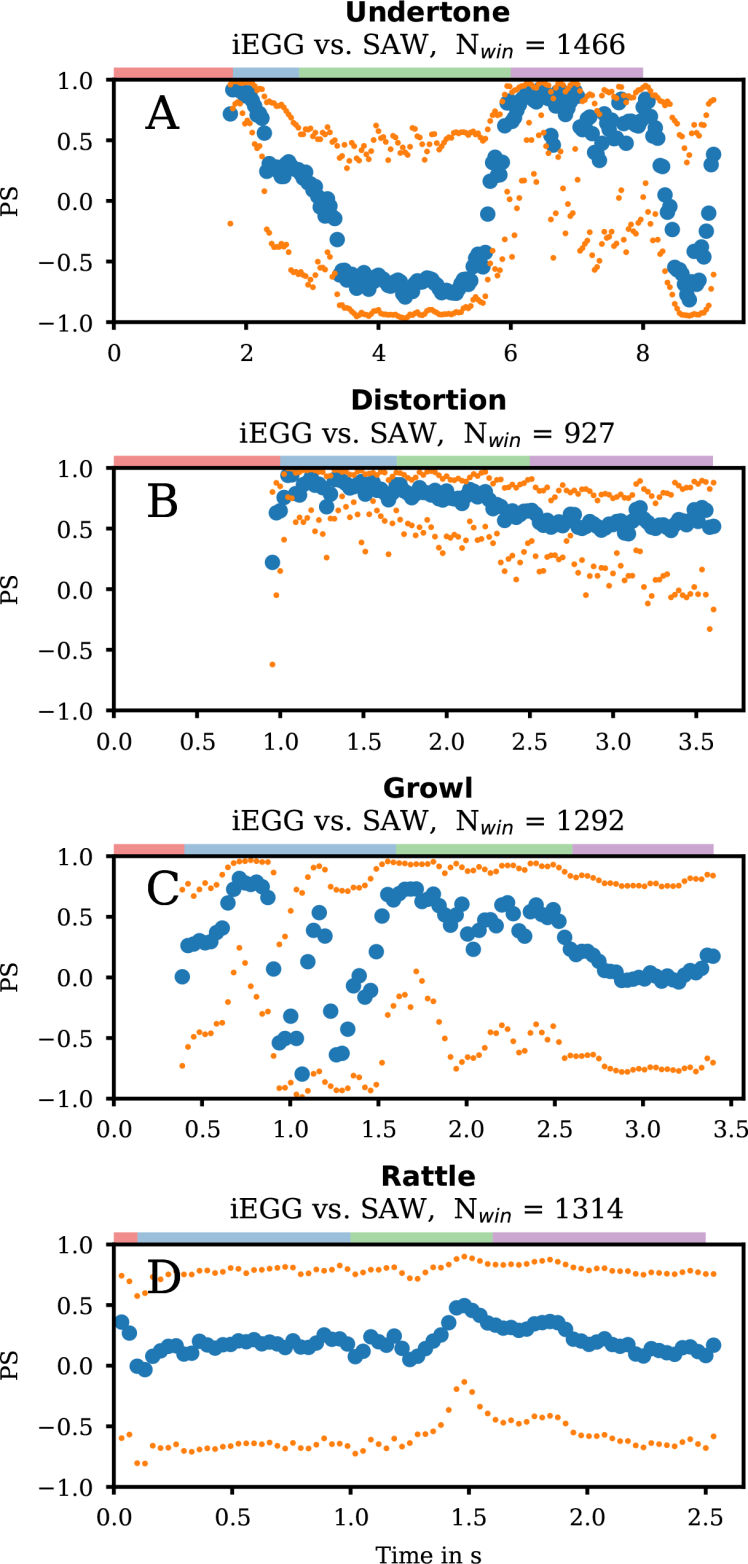
Phase synchronies for group 1. The median (blue dots) PS iEGG signal versus SAW signal for each analysed window of length *N*, as denoted in each subfigure, for the qualities Undertone (A), Distortion (B), Growl (C) and Rattle (D). The orange dots symbolize the ~25% and 75% quantiles of the data. At the top of each subfigure, the coloured rectangle denotes the region for clear phonation (red), low stable phonation (blue), glissando (green) and high stable phonation (purple). PS was not calculated for regions where the SAW could not be determined.

Furthermore, we analysed the phase relationship between VenF and VF oscillations for the entire task (figure 3A). During clear phonation, PS could not be computed, reasoned by a zero SAW signal. During low and high stable phonation, the iEGG and SAW signals were in-phase, as indicated by values of median PS close to 1. This relationship arises from an inherent nature of the signals: both the iEGG and SAW signal peaks during maximum glottal and supraglottic opening. Consequently, glottal and supraglottic oscillations are effectively in-phase, a finding consistent with visual observations from the videos and the iEGG-SAW comparison in figure 2A,E. However, it should be noted that the variability (distance between the 25% and 75% quantiles) is quite large, and, therefore, an exact determination of the phase relationship is only possible to a limited extent. As shown in figure 1A,E,I, and also acoustically perceived (electronic supplementary material, supplemental Undertone-hsdi_audio_glide.wav), the participant once again performed a slight downward glissando at the end of the task. Interestingly, this segment (approx. 8−10 s, see figure 3A) showed a decrease in synchronization similar to the upward glissando. A decrease in PS to approximately 0.3 can also be observed a few milliseconds before the glissando started, possibly due to a change in the VF oscillation characteristics.

Our observations support the hypothesis that fast, almost-periodic movements of the VF and VenF during Undertone phonation are coupled biomechanical oscillators [25]. Such oscillators can synchronize their periods through mutual coupling that actively controls $f_{\text{o}}$ and possibly controls synchronization even during dynamic transitions, like glissandi. However, during glissando, median PS first decreased and then shifted to nearly −1, indicating a shift from in-phase to a phase shift of 180° between the two signals. This shift may be related to a change in the temporal association between the maxima of the iEGG and SAW signals. During in-phase synchronization, the SAW maximum, representing the maximum supraglottic area, coincides with the iEGG maximum (i.e. minimum impedance, see figure 2A,E). During the glissando, however, the SAW maximum shifts to a position between the iEGG peaks (electronic supplementary material, figure S1), resulting in a phase shift of 180°. Nevertheless, the (slight) temporal precedence of VenF to VF contact persists in both in-phase synchronization (e.g. figure 2A,E) and a phase shift of 180° (electronic supplementary material, figure S1) of the iEGG and SAW signals.

#### 3.1.2. Distortion

Distortion is characterized by oscillation of the VF and VenF in an oscillation ratio of 1 : 1 (electronic supplementary material, video SV03_Distortion_A3.mp4 and SV04_Distortion_Db4.mp4). Supraglottic vibrations exhibit their largest amplitudes predominantly in the anterior region of the VenF. VF vibrations show complete closure of their vibrating parts.

The term ‘Distortion’ has also been referenced by authors within the framework of the voice school Complete Vocal Technique (CVT) [24]. In this context, Aaen *et al.* [24] performed a laryngoscopic examination that revealed notable inter-individual variance in observed laryngeal and vocal tract positioning. Congruently to our data and those from Caffier *et al.* [26], a medial–lateral and anterior–posterior narrowing of the supraglottic space is described there (electronic supplementary material, video SV03_Distortion_A3.mp4 and SV04_Distortion_Db4.mp4). In our data, the iEGG signal was largely unaffected during VenF vibration consistent with [24,26]. A reason for this observation was the small VenF vibrational amplitudes supported by our HSV analysis.

During low sustained phonation, we observed subharmonics with half of the initial $f_{\text{o}}$ of 216 Hz but low intensity for all four signals available (figure 1B,F,J,M and electronic supplementary material, supplemental Distortion-hsdi_audio_glide.wav). Furthermore, we observed no frequency jump at the beginning of low sustained phonation (approx. 1 s). This indicates a weak coupling of VenF and VF. At the beginning of glissando (at approx. 1.7 s), the subharmonics split, primarily prominent in the audio and GAW signal, indicating a further bifurcation (figure 1F,M) which persists during the rest of the whole task including high sustained phonation. Interestingly, further prominent subharmonics were present in the SAW (figure 1*j*) signal during high sustained phonation.

In contrast to Undertone, the SAW signal for Distortion is partly non-uniform, possibly due to artefacts. A reason might be the low tissue contrast when segmenting the VenF compared with the (partially visible) VF. Additionally, sometimes it spans two VF oscillations and exhibits varying curve configurations and amplitudes of its maxima and minima (electronic supplementary material, video SV03_Distortion_A3.mp4 and SV04_Distortion_Db4.mp4 and figure 2B,F).

However, considering the iEGG and GAW signal, changes in both impedance and contact area occur simultaneously during the closing phase. In contrast, the opening phase is initiated by an increase in impedance while vocal fold contact persists. This means that the contact area only begins to increase once the cranial portions of the vocal folds start to separate (figure 2B,F).

iEGG, GAW and SAW oscillations were predominantly in-phase (figures 2B,F and 3B). In contrast to Undertone phonation, the median PS only slightly changed during the glissando. Notably, Sakakibara *et al.* [21] describes a similar phonation type as a ‘pressed’ voice where the oscillators, similar to our data, show a phase shift of 180°.

In summary, similarities are evident between Undertone and Distortion, which suggest that Distortion may be a variant of vocal–ventricular–phonation. We thus hypothesize that the difference between the 1 : 1 and 1 : 2 synchronization is pitch-dependent, possibly related to the degree of VenF adduction.

#### 3.1.3. Growl

Growl phonation involves oscillation of the VF, the epiglottis, the arytenoid cartilages and the aryepiglottic fold. This supraglottic oscillation thus involves additional structures beyond the sole oscillation of the VenF observed in Distortion and Undertone. During Growl, VenF vibration is not visible because of the aryepiglottal narrowing. It may be that VenF medialization [27], as postulated in Undertone and Distortion, is not actively involved in this mechanism. Growl is characterized more by an anterior–posterior approximation of the epiglottis and the arytenoids, which covers the VF for most of the vibration (electronic supplementary material, video SV05_Growl_Eb3.mp4 and SV06_Growl_Bb3.mp4). We could not differentiate whether this movement was caused by the muscular components of the aryepiglottic fold (aryepiglottic sphincter muscle [28]) or by passive approximation of the epiglottis and the arytenoid cartilage conveyed through external structures. Also, we could not draw any conclusions about the VF oscillation due to the supraglottic occlusion.

Sakakibara et al. [7] describe Growl phonation in a similar way. Here, the described supraglottic oscillations are also induced by pronounced anterior–posterior compression, further supported by X-ray images demonstrating the retraction of the epiglottis. Also Aaen *et al.* [24] describe Growl phonation from an endoscopic examination as the vibration of the arytenoids against or adjacent to the epiglottis. In this respect, there is agreement with the available data. In contrast to [24] (no change) and [7] (elevation), visual analysis of our HSV data indicates a lowering of the larynx for the transition from clear phonation to Growl. A similar vocal tract configuration is observed in a Western-style classically trained singer [29]. This suggests that there are different possible mechanisms for achieving the epiglottis–arytenoid approximation.

Like Undertone, at the beginning of Growl at a time point of 0.4 s, we observed a small frequency jump of those harmonic in the audio signal from 157 to 161 Hz (figure 1C,G and electronic supplementary material, supplemental Growl-hsdi_audio_glide.wav). This may also indicate a destabilization of the initial limit cycle. For the lower sustained phonation (0.6–1.6 s) we observed subharmonics at approximately 77 Hz (table 1).

While in Undertone and Distortion, the supraglottic oscillator remains tightly entrained to the VF $f_{\text{o}}$ throughout pitch glides, Growl exhibits a divergent pattern. During glissando and high-pitched sustained phonation, the subharmonic frequency of the supraglottic structures remains approximately constant, resulting in a transition of the VF–VenF frequency ratio from 2 : 1 to 3 : 1 (figure 1C,G,K). This suggests that the natural frequency of VenF was not affected by the increase of $f_{\text{o}}$ of the VF.

The change of the entrainment ratio is consistent with theoretical models of flexible coupling between nonlinear oscillators [30,31]. Notably, the pitch perceived by the singer aligns with 2$f_{\text{o}}$ at low and 3$f_{\text{o}}$ at high phonation levels (156 and 233 Hz, respectively), underscoring a perceptual dominance of the VF sound source (table 1).

We propose that the insufficient resonance adjustment in the supraglottic structures impedes continuous synchronization during glissando. This contrasts with Undertone and Distortion, where persistent coupling implies ongoing resonance tuning. Nonetheless, in Growl, harmonic relations to integer ratios between the two oscillators are re-established during sustained phonation, suggesting re-entrainment at stable phonatory targets.

The SAW maximum slightly precedes distinctly reduced peaks of the iEGG signal (figure 2C,G), suggesting—similar to Undertone—a minimal temporal lead of the supraglottic oscillation relative to the VF oscillation. In contrast to Undertone and Distortion, the SAW signal reflects not only the movement of the VenF but also the joining supraglottic oscillators without differentiating between those. The SAW signal shows an intermediate minimum (at approx. 9 ms for Eb3 and at approx. 10 ms for Bb3, respectively). This might be related to an intermittent medial, closing motion correlated with the cranial movement of the mucosal wave through the epilaryngeal tube (electronic supplementary material, video SV05_Growl_Eb3.mp4 and SV06_Growl_Bb3.mp4). Such anterior–posterior constriction in the area of the epilaryngeal tube, as observed for Growl, is also described in articulation [32] and for certain singing styles [29,33], albeit to a lesser extent.

Similar to Distortion, at the beginning of the low sustained note, the median of PS between iEGG and SAW signal was partly synchronized and in-phase. The PS was slightly lower during glissando and high sustained phonation (figure 3C). Still, synchronization is less compared with Undertone and Distortion, in line with the reduced synchronization between VF and supraglottic vibration. Between approximately 0.9 and 1.6 s, however, a prominent decrease in PS occurs, which could be the result of a sudden instability of the mechanism (also visible in figure 1C,G,K at 1 s).

#### 3.1.4. Rattle

Rattle presents as a mechanism with oscillation of the VF, the VenF, the epiglottic petiole and the mucosa of the arytenoid cartilages and the aryepiglottic fold. It exhibits the highest differentiation of $f_{\text{o}}$ of VF from that of the supraglottic oscillator, evident in an oscillation ratio ranging from 4 : 1 to 7 : 1. In comparison with other qualities, the supraglottic configuration in Rattle differs due to a posterior constriction of the pharynx. Furthermore, *piriform sinuses* were partially occluded. If visible, we detected complete contact of the vibrating part of VFs, while the adduction of the *processus vocalis* appears to be incomplete (electronic supplementary material, video SV07_Rattle_Eb4.mp4 and SV08_Rattle_Bb4.mp4).

To our knowledge, no data on that type of voice quality using HSV are available in the literature. Interestingly, [24] and [26] described a similar supraglottic configuration in prior endoscopic explorations of Rattle phonation. They found an anterior–posterior and medial narrowing in combination with laryngeal elevation. This leads to a significant reduction or collapse of the *piriform sinuses* (in contrast to all other voice qualities in group 1) and is similar to that of other voice qualities used in contemporary commercial music like Belting and Twang [29]. Aaen *et al.* [24] describes Rattle as a vibration of the mucosa of the arytenoid cartilages against or adjacent to one another [24]. Caffier *et al.* [26] proposes that Rattle shows a smacking of the mucosa in the region of the cuneiform and corniculate cartilages on the epiglottis. In our observations, VenF vibrations are transitioning through the supralaryngeal tube, including the arytenoids’ mucosa, the aryepiglottic fold and the mucosa of the epiglottic petiole of the epiglottis (but not the whole epiglottis in contrast to Growl).

For Rattle, we found the most complex behaviour of glottal and supraglottic oscillations for group 1 for iEGG, audio and SAW signals (figure 1D,H,I and electronic supplementary material, supplemental Rattle-hsdi_audio_glide.wav). Besides a frequency jump of the harmonic close to the perceived pitch of 311 Hz of the audio signal from 263 to 305 Hz, we detected subharmonics as the singer starts with Rattle at 0.1 s. During glissando (1.0–1.6 s) multiple frequency splits occurred, looking like a honeycomb structure. This indicates that biphonation remaining present at high sustained phonation. Resonances of the vocal tract could play a role in the prominent appearance of the harmonics around 300–400 Hz in the audio signal (figure 1D,H) that is in congruence to the perceived pitch of 311–466 Hz (table 1).

The almost periodic iEGG signal shows additional periodic variations of the signal envelope (figure 2D,H). In addition to rapid waveforms, which correlate with the subjectively perceived pitch and originate from the VF oscillation, a superimposition of a low frequency was observed for iEGG, contributing to periodic movements of the signal baseline.

Furthermore, the almost periodic SAW signal correlates with the slow modulation of the iEGG waveforms. Notably, the maximum of the superimposed envelope in the iEGG signal does not coincide temporally with the maximums of the SAW, indicating a phase shift between the VF and the VenF. This finding is supported by the median PS of the iEGG versus SAW (figure 3D), indicating overall low synchrony. More detailed, the minimum of the SAW signal does not necessarily have to exclusively determine the temporal location of the minimum of the iEGG envelope signal. It seems, that the minimum of the envelope in the iEGG signal precedes the SAW minimum.

In sum, for Rattle, we found similarities to Undertone and Distortion (involving VenF and VF oscillation) and Growl (increase of anterior–posterior approximation and lack of simultaneous adaptation of the supraglottic oscillator to the $f_{\text{o}}$ of VF during glide. The additional pharyngeal constriction in Rattle mentioned above is unique for group 1.

### 3.2. Group 2

In contrast to the almost periodic oscillating mechanisms of group 1, the oscillating mechanisms of group 2 showed an increase in irregularity in the glottal and supraglottic vibration. These mechanisms showed various irregular supraglottic vibration patterns with or without glottal oscillations (if visible).

#### 3.2.1. Group 2a

Group 2a combines irregular mechanisms where the glottis is visible. The mechanisms categorized here mainly differ in the degree of glottal adduction, while supraglottic oscillations play a secondary role. For these voice qualities, it was not possible to segment a SAW due to the low contrast difference between VF and VenF. Consequently, we could not calculate PS.

##### 3.2.1.1. VocalFry

VocalFry involves VF vibration patterns with a long closed phase and a small intermediate oscillation in the open phase just before closing (electronic supplementary material, video SV09_VocalFry.mp4). In contrast to the mechanisms described before, VenF oscillation was limited to small aperiodic motions of the epithelium (electronic supplementary material, video SV09_VocalFry.mp4) leading us to the assumption that these small amplitudes do not determine the acoustic outcome to a large extent.

VocalFry was labelled as M0 by Roubeau et al. [34] and their description is confirmed by our data. Herzel *et al.* hypothesized that VocalFry is associated with de-synchronization of horizontal movement of the VF body and vertical movement of the lax cover [35]. Furthermore, VocalFry involuntary occurs in patients with functional dysphonia and healthy speech production [36–40] but is also part of vocal effects in artistic settings.

**Figure 4.**
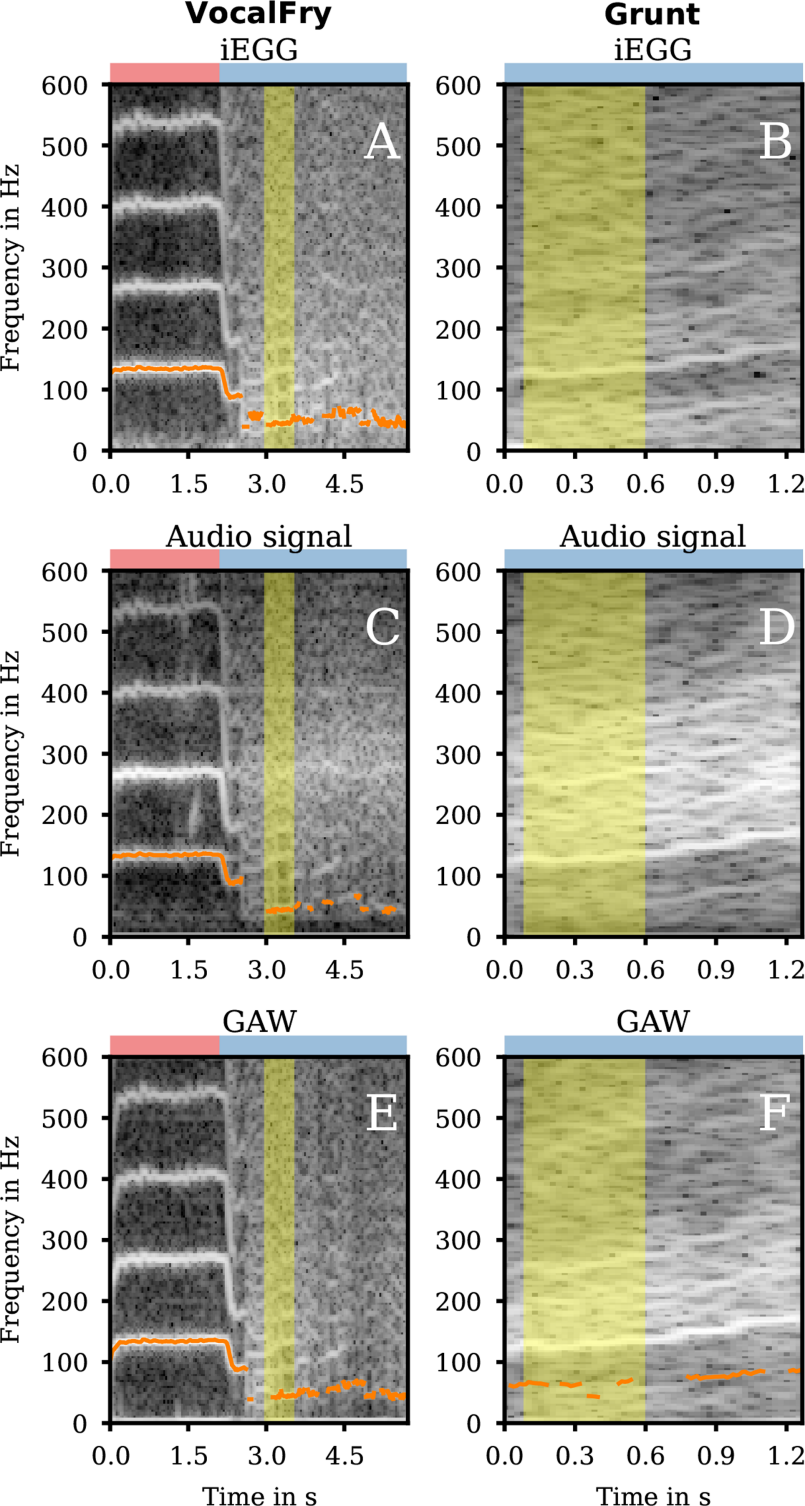
Spectrograms for group 2a. The spectrograms of the iEGG signals (A, B), the audio signals (C, D) and the GAWs (E, F) for the qualities VocalFry (1st column) and Grunt (2nd column) are shown in grey colour. The $f_{\text{o}}$ trajectories are symbolized in orange. At the top of each subfigure, the coloured rectangle denotes the region for clear phonation (red) and irregular phonation (blue). The areas highlighted in yellow correspond to the segments of sustained phonation depicted in the electronic supplementary material videos and analysed for their $f_{\text{o}}$ in table 1.

In our data, after the onset of VocalFry at approximately 2 s (figure 4A,C,E and elelctronic supplementary material, VocalFry-hsdi_audio_glide.wav), $f_{\text{o}}$ of the audio signal rapidly decrease from approximately 136 to 88 Hz and then overlapped by multiple subharmonics of low amplitude. The VF oscillation produced distinct glottal pulses with low $f_{\text{o}}$ ($f_{\text{o}}=44$ Hz in the iEGG, audio and GAW signal, see figure 4A,C, electronic supplementary material, VocalFry-hsdi_audio_glide.wav, and table 1).

**Figure 5.**
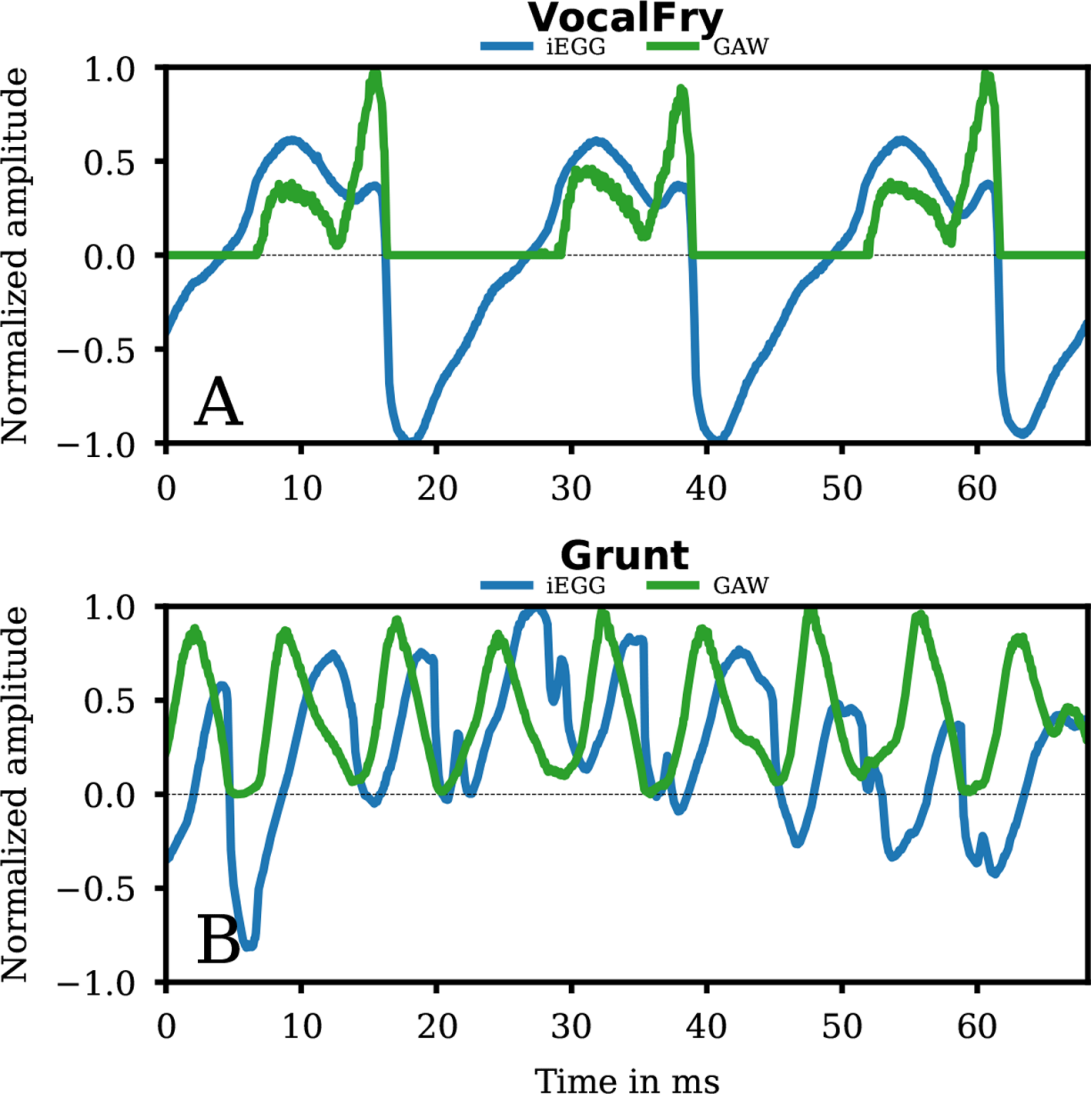
Detailed segments of signals of group 2a. The iEGG and GAW signals as denoted in the legends for the qualities VocalFry (A) and Grunt (B).

The GAW signal was characterized by an elongated closed phase (figure 5A). In the open phase, the GAW signal shows a double peak with a minimum reaching the value close to zero (at approx. 12, 36 and 58 ms). This observation correlates with the principal shape of the iEGG in that region, indicating that iEGG was primarily determined by VF oscillations. Thus, VocalFry correlated with the description of e.g. [26,41–44].

Cielo *et al.* [45] summarizes the available literature on muscular activation for VocalFry and describes it as a predominant action of the thyroarytenoid muscles confirmed by simulations [46]. Interestingly, no correlation between $f_{\text{o}}$ and VF length was described in VocalFry (overall, VF length was lower in VocalFry, correlating well with low cricothyroid (CT) and high transversus abdominis (TA) activity), suggesting a different mechanism for $f_{\text{o}}$ control as compared with chest and modal register [47] and supported by the impression of the singer in our study. Our singer could not address a specific pitch and could alter $f_{\text{o}}$ only in a limited region.

##### 3.2.1.2. Grunt

Grunt is characterized by reduced VF adduction, resulting in irregular oscillations of VF and supraglottic structures (VenF and aryepiglottic fold) (electronic supplementary material, video SV10_Grunt.mp4). Generally, the VF closure was absent or fleeting or only sporadically present. The regular pattern of mucosal wave movement during VF vibration (alternating convergent–divergent geometry shape of the glottis [48]) was disturbed. Normally, VF vibration displays a typical opening and closing pattern from caudal to cranial, characterized by a phase shift between the lower and upper edges of the vocal folds [49]. In contrast, Grunt exhibits only a localized contact of both VF, such as at the anterior or posterior third, without being integrated into the typical oscillatory pattern. Other irregular behaviour was visible as an additional VF vibration without closure (represented in a GAW double peak, e.g. at 60−70 ms in figure 5B) or VF left–right asymmetry (electronic supplementary material, video SV10_Grunt.mp4). Our observations were similar to endoscopic findings for Grunt by Aaen *et al.* [24].

The spectrograms of the iEGG, audio and GAW signal (figure 4B,D,F and electronic supplementary material, supplemental Grunt-hsdi_audio_glide.wav) showed a slight increase in $f_{\text{o}}$ starting at 125 Hz overlaid with a non-negligible noise floor of unknown origin. Glottal and supraglottic configuration suggests high trans-glottal airflow corresponding to desynchronized oscillations in *ex vivo* simulations [8].

### 3.2.2. Group 2b

The mechanisms described in this section are characterized by aperiodic vibratory movements of the supraglottis (according to [2,6]), resulting from various types of supraglottic sphincter activation in combination with or without glottal adduction.

#### 3.2.2.1. DeathGrowl

For DeathGrowl, we found aperiodic vibrations of the entire epiglottis, arytenoid cartilages and aryepiglottic folds (electronic supplementary material, video SV11_DeathGrowl.mp4). The anteroposterior adduction between the epiglottis and arytenoid cartilages is similar to Growl phonation, indicating possible aryepiglottic sphincter activation. Because of a hidden glottis, we found no direct evidence for laryngeal oscillations.

To our knowledge, no studies in the scientific literature used HSV to describe a comparable artistically used voice or sound production mechanism. This vocal technique, used in Death Metal, achieves a desired roughness in sound.

**Figure 6.**
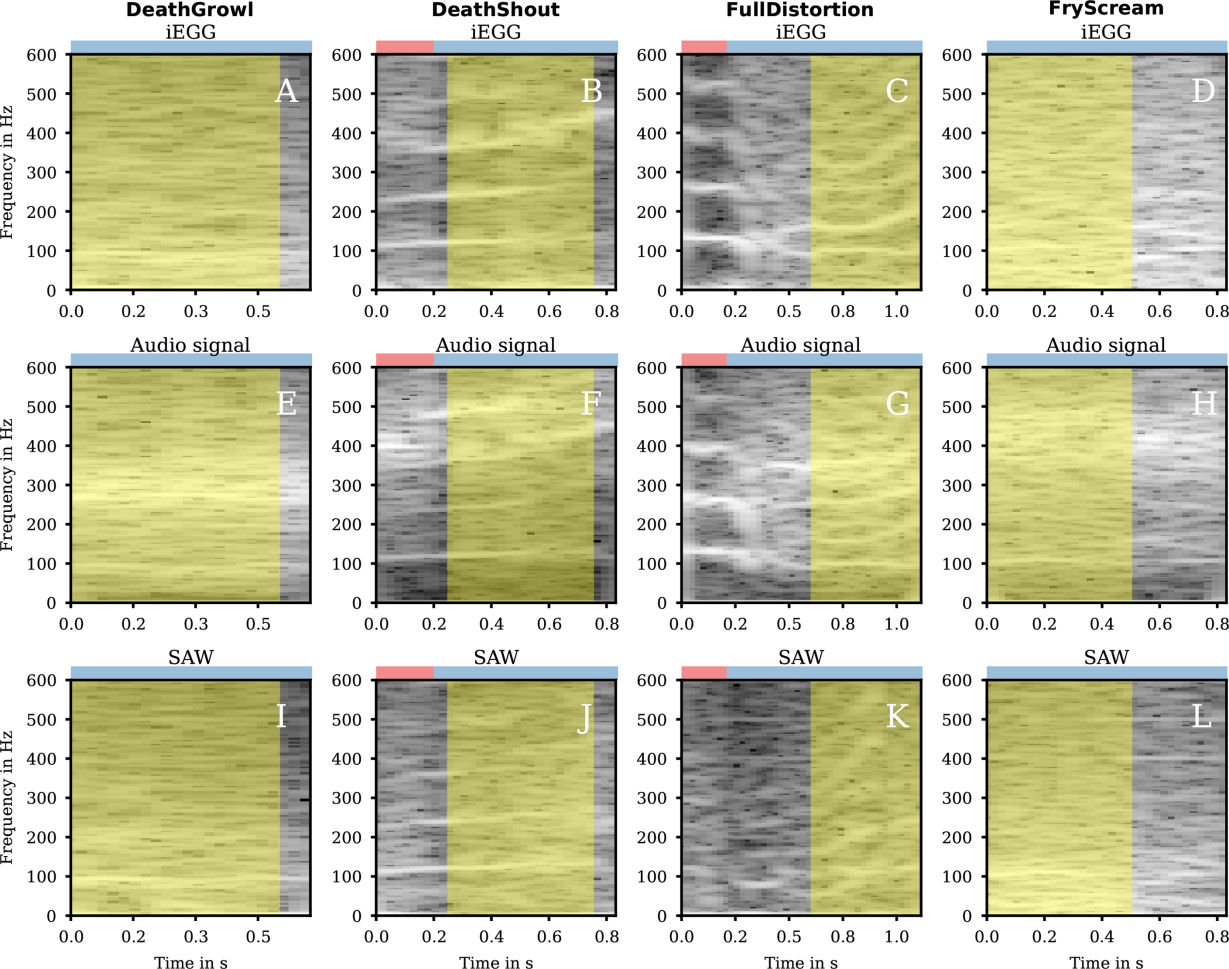
Spectrograms for group 2b. The spectrograms of the iEGG signals (A–D), the audio-signals (E–H) and the SAWs (I–L) for the qualities DeathGrowl (1st column), DeathShout (2nd column), FullDistortion (3rd column) and FryScream (4th column) are shown in grey colour. At the top of each subfigure, the coloured rectangle denotes the region for clear phonation (red) and irregular phonation (blue). The areas highlighted in yellow correspond to the segments of sustained phonation depicted in the electronic supplementary material videos and analysed for their $f_{\text{o}}$ in table 1.

In the spectrograms (figure 6A,E,I and electronic supplementary material, supplemental DeathGrowl-hsdi_audio_glide.wav), we observed an $f_{\text{o}}$ around 100 Hz for iEGG, audio and SAW signal. However, because of very low intensity, it was only extracted for the SAW signal by the used algorithm (table 1).

**Figure 7.**
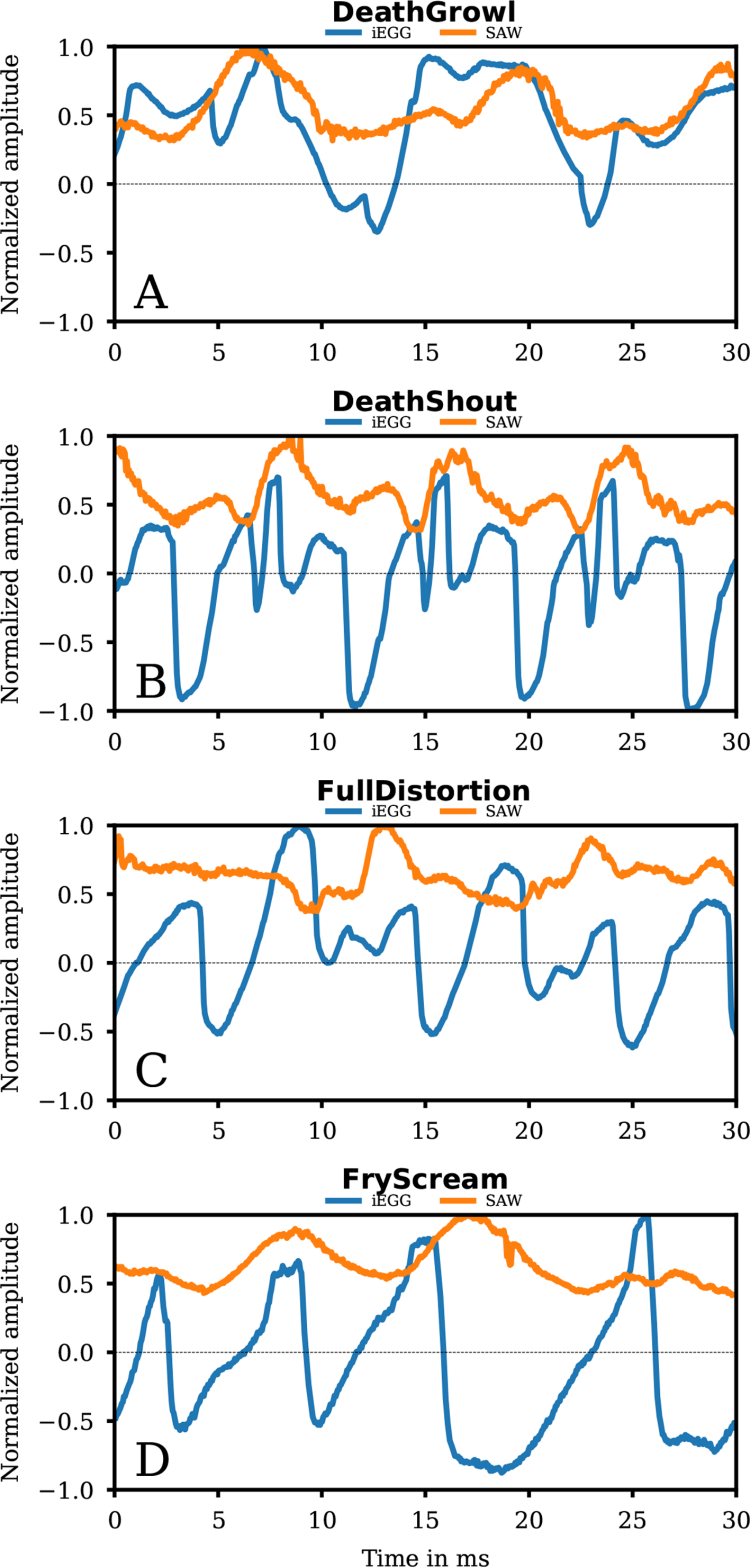
Detailed segments of signals of group 2b. The iEGG and SAW signals as denoted in the legends for the qualities DeathGrowl (A), DeathShout (B), FullDistortion (C) and FryScream (D).

**Figure 8.**
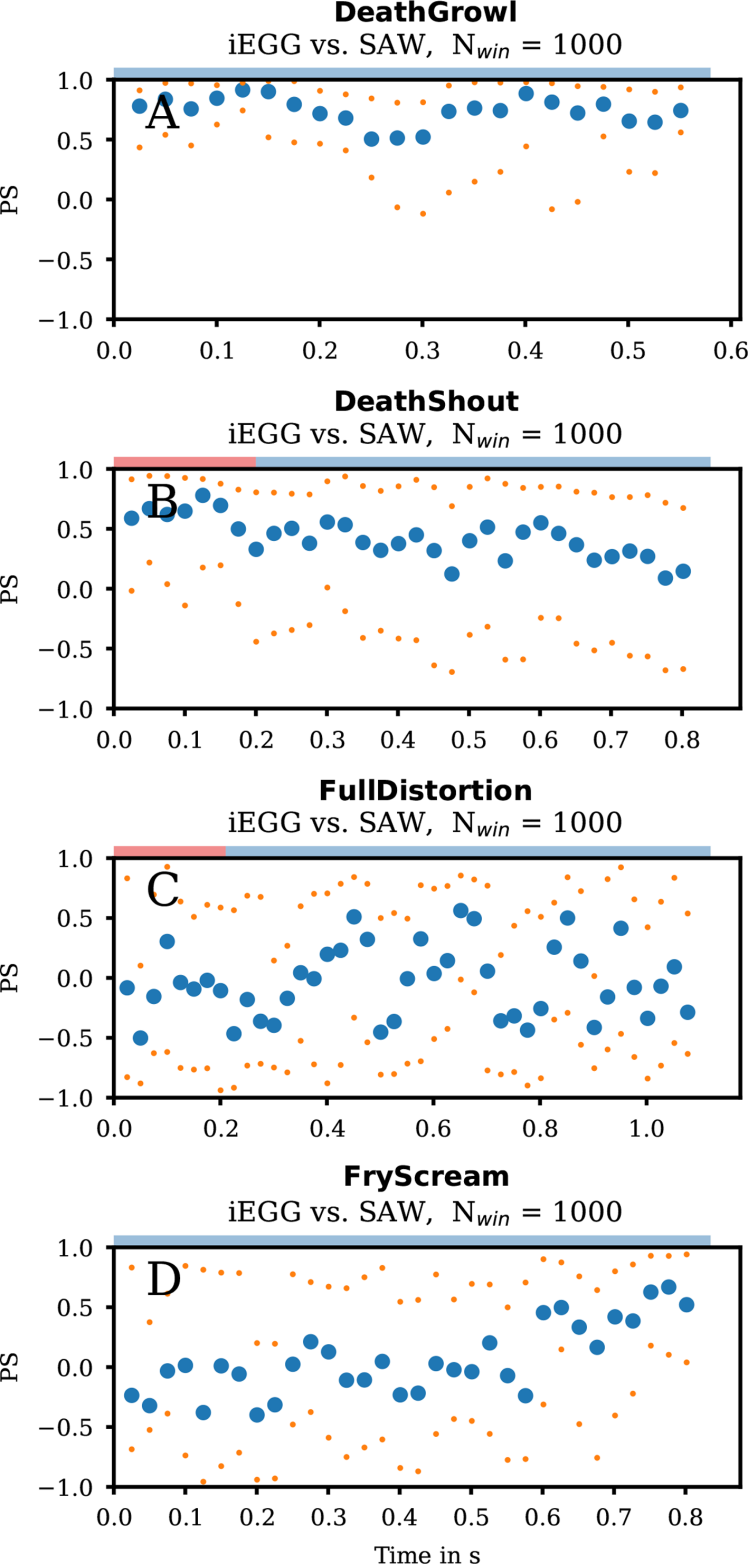
Phase synchronies for group 2b. The median (blue dots) PS iEGG versus SAW signals for each analysed window of length *N*, as denoted in each subfigure, for the qualities DeathGrowl (A), DeathShout (B), FullDistortion (C) and FryScream (D) are coloured as denoted in the legends. The orange dots symbolize the ~25% and ~75% quantiles of the data. At the top of each subfigure, the coloured rectangle denotes the region for clear phonation (red) and irregular phonation (blue).

In the iEGG signal, no patterns of normal VF oscillation are present (figure 7A). However, the iEGG variations show a correlation with the SAW signal as indicated in figure 8A. This might indicate the absence of actual VF oscillation, potentially due to a lack of VF adduction. iEGG and SAW signals were (mostly) in-phase, probably due to the dominant supraglottic movement.

From a voice pedagogic perspective, co-author D.P. describes DeathGrowl phonation as combining Grunt with supraglottic adduction similar to Growl, but, due to the hidden glottis, we cannot visually prove or refute laryngeal oscillation. However, the analysis confirms the absence of regular VF oscillation in the iEGG signal.

#### 3.2.2.2. DeathShout

DeathShout exhibits aperiodic vibration of the entire epiglottis, arytenoid cartilages and aryepiglottic folds (electronic supplementary material, video SV12_DeathShout.mp4).

Bailly *et al.* described similar mechanisms involving aperiodic VenF/VF vibration during ‘shouts’, defined as phonation with a louder voice, highly stable phonation and perceptible vocal effort, which introduced strong roughness in the vocal output [2]. In a physical model, Matsumoto *et al.* have shown that powerful supraglottic adduction increases the supraglottic $f_{\text{o}}$ and chaotic desynchronized dynamics [50].

In contrast to DeathGrowl, DeathShout (figure 7B) shows a disturbed but periodic pattern of the iEGG signal. An increased tonality can also be perceived acoustically (electronic supplementary material, supplemental DeathShout-hsdi_audio_glide). However, the iEGG signal seems superimposed by supraglottic oscillation probably correlated with the SAW signal. Still, iEGG and SAW phases are less synchronized compared with DeathGrowl (figure 8B).

The supraglottic narrowing mechanism presented for DeathShout seems to be comparable to Growl and DeathGrowl. Thus, the vibration mechanism involves the entire epiglottis and arytenoid cartilages. Visible portions of the *piriform sinuses* indicate that pharyngeal constriction is not maximal.

#### 3.2.2.3. FullDistortion

For FullDistortion, we observed an aperiodic vibration of the entire epiglottis, the aryepiglottic fold and the arytenoid mucosa (electronic supplementary material, video SV13_FullDistortion.mp4). FullDistortion was different from DeathShout and DeathGrowl by its pharyngeal configuration. Here, an additional pharyngeal constriction occurs combined with an enormous laryngeal elevation and anterior–posterior adduction of arytenoids and epiglottis. Thus, the pharyngeal configuration resembles Rattle (group 1) but with a higher degree of constriction. The larynx is being squeezed from the outside by the pharyngeal wall, and the *piriform sinuses* collapse entirely, as far as can be assessed endoscopically.

The spectrograms of all signals show an irregular, noisy pattern, indicating inhomogeneities in the underlying mechanism (figure 6C,G,K and electronic supplementary material, supplemental DeathShout-hsdi_audio_glide.wav). Therefore, for FullDistortion, a valid automatic $f_{\text{o}}$ detection was not successful. Visually, at approximately 0.4 s, a bifurcation occurs in audio and iEGG signal, splitting from approximately 110 to 100 and 150 Hz. Whereas the latter undergoes a glissando starting at approximately 0.8 s, which is also visible in the SAW spectrogram (figure 6K) and might thus be of supraglottic origin, the first keeps constant.

The iEGG signal is moderately regular with slight changes in amplitudes (figure 7C). Moreover, the maxima of the (more irregular) SAW signal correlate with the instants of reduced iEGG peaks. A reason for this observation could be an impedance reduction caused by the lateral movement of the supraglottic oscillator during oscillation. The overall behaviour of PS of iEGG and SAW signal fluctuates significantly over the whole task, emphasizing its heterogeneity (figure 8C).

#### 3.2.2.4. FryScream

For FryScream, we found a small vibration amplitude compared with the other group 2b mechanisms (electronic supplementary material, video SV14_FryScream.mp4) which could be related to an increase in pharyngeal constriction. Supraglottic vibration affects only the mucosa of the arytenoids (electronic supplementary material, video SV14_FryScream.mp4).

Similar to DeathGrowl, for FryScream we visually observed an $f_{\text{o}}$ around 100 Hz for iEGG, audio and SAW signal (figure 6D,H,L). While the auditory impression is also comparable, FryScream may subjectively be perceived as ’brighter’ (electronic supplementary material, supplemental DeathShout-hsdi_audio_glide.wav). Because of very low intensity, it could not automatically be computed by the used algorithm (table 1).

In detail, the iEGG and SAW signals are irregular and randomly arranged regarding their maxima (figure 7D). Indeed, the general pattern of the iEGG signal for FryScream resembles that of VocalFry but varies significantly in period length. Co-author D.P. describes VocalFry as the ‘laryngeal mechanism’ in FryScream. As the glottis is not visible in our data, we can only hypothesize (based on the iEGG and SAW signals) that the VF performs aperiodic oscillations desynchronized with the supraglottis supported by PS in figure 8D. We found a slight change in PS that starts in asynchrony and tends to be somewhat synchronized and in-phase. The latter finding suggests that VF and supraglottic oscillation become more aligned as FryScream progresses.

### 3.3. Limitations

Our study encompasses only a single subject. Therefore, extrapolation to a larger cohort is limited.

Our HSV data provide an endoscopic perspective of the oscillating structures, not allowing the quantification, i.e. of absolute values of glottal and/or supraglottic areas. Due to unfavourable contrast in the pictorial data, segmentation is prone to artefacts for some voice qualities, e.g. overlapping motions. It should also be noted that the SAW (and GAW) signals cannot provide information about the contact areas. Thus, the minimum of the SAW does not necessarily correspond to the point of maximal supraglottic contact. This means that supraglottic oscillations also influenced the iEGG signal, and, in some cases, we observed a temporal offset between iEGG and SAW. The effect is more apparent when the portion of supraglottic vibrating structures increases. Additionally, we cannot preclude a technically induced shift, as described by Hampala *et al.* [14]. However, the high correspondence between the GAW and EGG signals (if available) suggests no substantial technical shift in our data.

To our best, we positioned the EGG electrodes at the level of the VF. However, during these extreme vocal tasks, we cannot exclude a variability in the proportions and ratio of the VF and VenF impedances.

Furthermore, audio and EGG signals were recorded at 20 kHz in alignment with the image data without using an analogue low-pass filter to exclude aliasing effects. However, we believe this does not affect the data presented here.

## 4. Conclusions

We successfully differentiated 10 distinct voice qualities into almost periodic and aperiodic oscillators. We demonstrate that it is possible to recruit various supraglottic oscillators that can behave synchronously or asynchronously with VF vibration and exhibit both almost periodic and aperiodic behaviour. Excessive as well as insufficient glottal or supraglottic adduction leads to asynchronous vibrations. In contrast, an intermediate supraglottic position supported nearly periodic vibration processes and oscillations with various frequency ratios.

The degree and localization of supraglottic adduction may play a role in varying coupling strength of glottal and supraglottic oscillators: as shown in computational and physical models [8,12,51], the degree of VenF adduction in the transglottal flow seems to determine the transition of weaker synchronization with an oscillation ratio 1 : 1 and anterior VenF oscillation (Distortion) to robust synchronization with an oscillation ratio 2 : 1 and complete VenF oscillation (Undertone) in our *in vivo* data. VenF adduction is conducted by tensing and shortening the muscle fibres of the thyroarytenoid and ventricular muscles located lateral to the glandular and fatty structure [27] in the posterior part of the VenF. In contrast, the anterior part of the VenF is nearly free of muscle fibres [52]. Thus, it can be concluded that, for a trained singer, it is possible to consciously target and maintain a specific VenF distance (also during the glissando). A close spatial proximity of the oscillators indicates that mechanical coupling is conceivable. Moreover, aerodynamic coupling is also a possible coupling mechanism.

Growl and Rattle introduce another level of supraglottic vibrations caused by a pronounced anterior–posterior constriction. For these two qualities, the synchronization decreases during the glissandi as the vibrating mass increases compared with vocal–ventricular–phonation mechanisms. However, frequency locking occurs at integer multiples of the $f_{\text{o}}$ of VF during sustained phonation. During the adjustment of $f_{\text{o}}$ of VF in glissandi, nonlinear phenomena such as bifurcations and biphonation occur. In contrast, aperiodic vibrations occur by an increase in trans-glottal airflow (observed for Grunt) or are related to strong glottal (observed for VocalFry) or supraglottic (observed in all other qualities) adduction. Here, our data confirm the findings of Inoue *et al.* evaluated in simulations [8].

Several mechanisms are known to produce chaos in the context of human voice production solely in VF vibration like left–right asymmetry [53], anterior–posterior modes [54], excessively high subglottal pressure and acoustical coupling with supraglottic resonances [55]. For a recent review, see also [30]. In our study, we could expand this list with aperiodic glottal and supraglottic vibrations.

Regarding supraglottic constriction, for DeathGrowl and DeathShout, we found a closer positioning of the epiglottis and the arytaenoid cartilages. FullDistortion and FryScream additionally exhibit laryngeal impingement due to pharyngeal constriction. However, this is much more pronounced in FryScream and combined with laryngeal elevation.

Our results could be a basis for the work with patients to develop a supraglottic substitute voice. Knowledge of the differentiated activation of VenF and the aryepiglottic or pharyngeal sphincter might be beneficial here. It could also assist in differentiated musical applications by targeting specific sounds desired by the artist. Here, particular changes in vocal tract configuration might also be beneficial for targeting specific irregular mechanisms. However, while such changes cannot derived from HSV data, they could be more precisely examined using three-dimensional magnetic resonance imaging (MRI) data of the vocal tract, which could be a beneficial next step to explore.

## Data Availability

All measured data and Python scripts used for analysis, computation and visualization are in the supplemental section. Supplementary material is available online [56].
